# Barrier crossing in small avian migrants: individual tracking reveals prolonged nocturnal flights into the day as a common migratory strategy

**DOI:** 10.1038/srep21560

**Published:** 2016-02-15

**Authors:** Peter Adamík, Tamara Emmenegger, Martins Briedis, Lars Gustafsson, Ian Henshaw, Miloš Krist, Toni Laaksonen, Felix Liechti, Petr Procházka, Volker Salewski, Steffen Hahn

**Affiliations:** 1 Department of Zoology, Palacký University, tř. 17. listopadu 50, CZ-771 46 Olomouc, Czech Republic; 2 Museum of Natural History, nám. Republiky 5, CZ-771 73 Olomouc, Czech Republic; 3 Department of Bird Migration, Swiss Ornithological Institute, Seerose 1, CH-6204 Sempach, Switzerland; 4 Department of Animal Ecology/Ecology and Genetics, Uppsala University, Norbyvägen 18D, SE-75236, Uppsala, Sweden; 5 Section of Ecology, Department of Biology, University of Turku, FI-20014, Turku, Finland; 6 Institute of Vertebrate Biology, Academy of Sciences of the Czech Republic, Květná 8, CZ-603 65 Brno, Czech Republic; 7 Michael-Otto-Institut im NABU, Goosstroot 1, D-24861 Bergenhusen, Germany

## Abstract

Over decades it has been unclear how individual migratory songbirds cross large ecological barriers such as seas or deserts. By deploying light-level geolocators on four songbird species weighing only about 12 g, we found that these otherwise mainly nocturnal migrants seem to regularly extend their nocturnal flights into the day when crossing the Sahara Desert and the Mediterranean Sea. The proportion of the proposed diurnally flying birds gradually declined over the day with similar landing patterns in autumn and spring. The prolonged flights were slightly more frequent in spring than in autumn, suggesting tighter migratory schedules when returning to breeding sites. Often we found several patterns for barrier crossing for the same individual in autumn compared to the spring journey. As only a small proportion of the birds flew strictly during the night and even some individuals might have flown non-stop, we suggest that prolonged endurance flights are not an exception even in small migratory species. We emphasise an individual’s ability to perform both diurnal and nocturnal migration when facing the challenge of crossing a large ecological barrier to successfully complete a migratory journey.

Twice a year billions of birds undertake a migratory journey of several thousand kilometres to their non-breeding sites and back. The Palearctic-African flyway represents probably the largest avian migration system on earth^1^. An estimated 2.1 billion songbirds and near-passerines move from Europe to Africa each autumn^2^. At some point in time, nearly all of them have to cross a major ecological barrier, the 1500–2000 km wide Sahara Desert.

A crucial, and to date debated, issue is which migratory strategy individual songbirds use to reach their destination. In his seminal work, Moreau^3^ suggested “it does seem that an ability to maintain flight for 50–60 hours without food or water is essential for those birds which regularly migrate across the Sahara”. However, since the 1980s, an accumulating number of studies brought evidence in favour of the alternative intermittent migratory strategy^4^,^5^,^6^,^7^. In this scenario songbirds cross the Sahara in small steps: flying at night and resting and/or refuelling during the day. The picture, however, seems to be complex, as radar studies from various sites across the globe have brought evidence of nocturnal migrants regularly prolonging flight into the day when crossing large-scale barriers^8^,^9^,^10^,^11^. The proportion of diurnal migratory traffic in these studies was just a fraction of typical night-time migration, suggesting that landing occurs shortly around sunrise and that only some birds are capable of prolonged daytime flights. This means that some individuals show flexible migratory behaviour and they can switch from nocturnal to partially diurnal migration. In general, nocturnal migration is the prevailing pattern in small birds within the Palaearctic African migration system^12^. With our own survey we estimate that about 63% of species (44 out of 70 trans-Saharan migrants for which we have collated data; Supplementary Table S1) are expected to migrate during the night when crossing continental Europe. Another 16% migrate solely during the daytime, so the pool of individuals that might theoretically switch to temporary daytime migration is considerable. While we already know that daytime migration and prolonged flights across vast barriers do happen in small songbirds^13^, the magnitude, temporal and seasonal (spring vs. autumn passage) effects at the individual and species level remain little known for the large barriers between Europe and Africa.

Recent technical development of satellite transmitters and small light intensity data loggers (geolocators) has enabled research showing that at least in some larger migratory species, extreme endurance flights of several thousands of kilometres are possible^14^,^15^,^16^. Here we tracked several small songbird species, weighing about 12 g, and for the first time investigated their individual migratory patterns while crossing the Sahara Desert and the Mediterranean Sea. By the analysis of anomalies in the light patterns recorded during barrier crossing periods we aim to evaluate i) the occurrence and the timing of prolonged flights into the day by typical nocturnal migrants, ii) the day-to-day diversity in migratory patterns, and iii) to compare autumn and spring migration periods.

## Results

### Spatial and temporal occurrence of light anomalies

**Figure 1:**
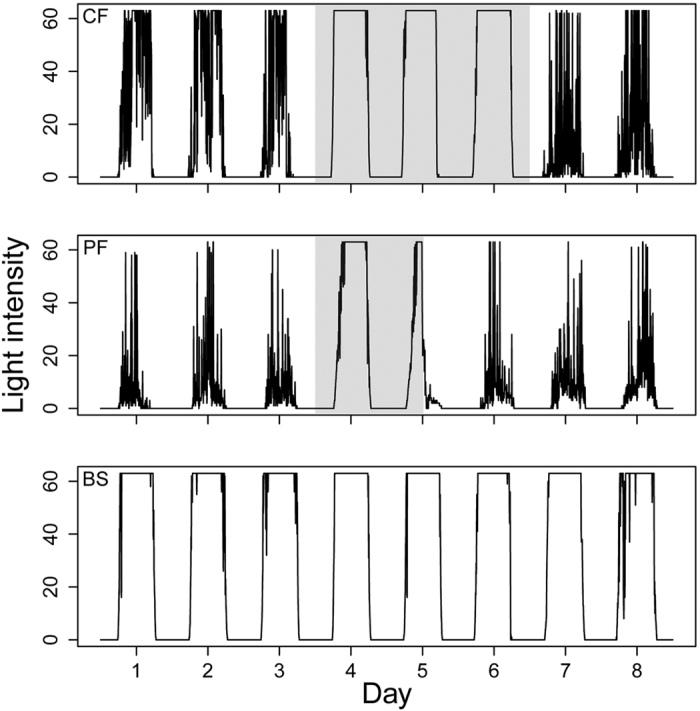
Representative examples of full light pattern anomalies (FLP, grey area) in a collared flycatcher (CF), pied flycatcher (PF) and barn swallow (BS) recorded by light-level geolocators on days while crossing the Sahara desert. The middle plot (PF) depicts an example of FLP that abruptly ends on the second day at 12:00 UTC. Tick marks on x-axis denote noon. The zigzag light pattern (shading caused by habitat and bird behaviour) a few days before and after the full light pattern in collared and pied flycatchers represents typical geolocator data for most birds throughout their annual cycle. The barn swallow data represent typical geolocator data for an aerial forager.

**Figure 2:**
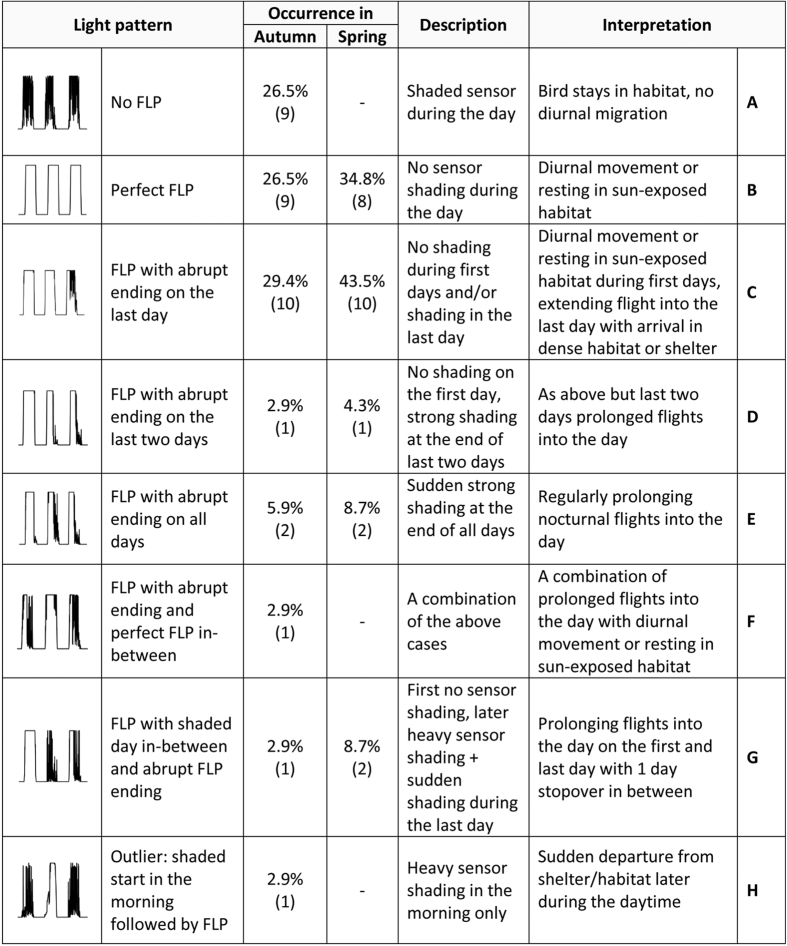
Detailed seasonal overview of daytime light pattern anomalies (FLP) recorded by geolocators while the birds were crossing the Sahara Desert. Each category is accompanied by a representative figure of recorded light intensities, % of occurrence (numbers of individuals are in parentheses), description of the anomaly and our most plausible interpretation (categories A–H).

All four species in this study, i.e. the collared flycatcher *Ficedula albicollis*, pied flycatcher *Ficedula hypoleuca*, Eurasian reed warbler *Acrocephalus scirpaceus* and aquatic warbler *Acrocephalus paludicola* (see Supplementary Table S2 for details) are widely thought to migrate at night and rest during daytime. This behaviour is reflected in geolocator data with a zigzagging light pattern during the day, caused by variable exposure of the light sensor during foraging, preening, resting and moving through vegetation (Fig. 1). In both spring and autumn migration periods, however, there was a noticeable pattern of continuous full light intensity (full light pattern; FLP) during the daytime in all four species, i.e. the sensor recorded maximum light levels for uninterrupted periods of several hours (for an overview of FLP anomalies see Fig. 2). This FLP anomaly lasted for 1–3 days. In the collared flycatcher it occurred in 12 out of 13 birds in autumn and in all birds in spring (11 individuals for which data have been recorded until spring). All pied flycatchers showed FLP in both autumn and spring (4 and 2 individuals, respectively). Similarly, all aquatic warblers had FLP (5 and 2 individuals). In reed warblers FLP occurred in 4 out of 12 birds in autumn and in all 8 birds tracked in spring. Summarising across species, all individuals showed FLP in spring and the absence of FLP was observed in autumn only (9 out of 34 tracked individuals). Seven of 9 birds without FLP still had a distinct zigzag pattern of increased light intensities.

**Figure 3:**
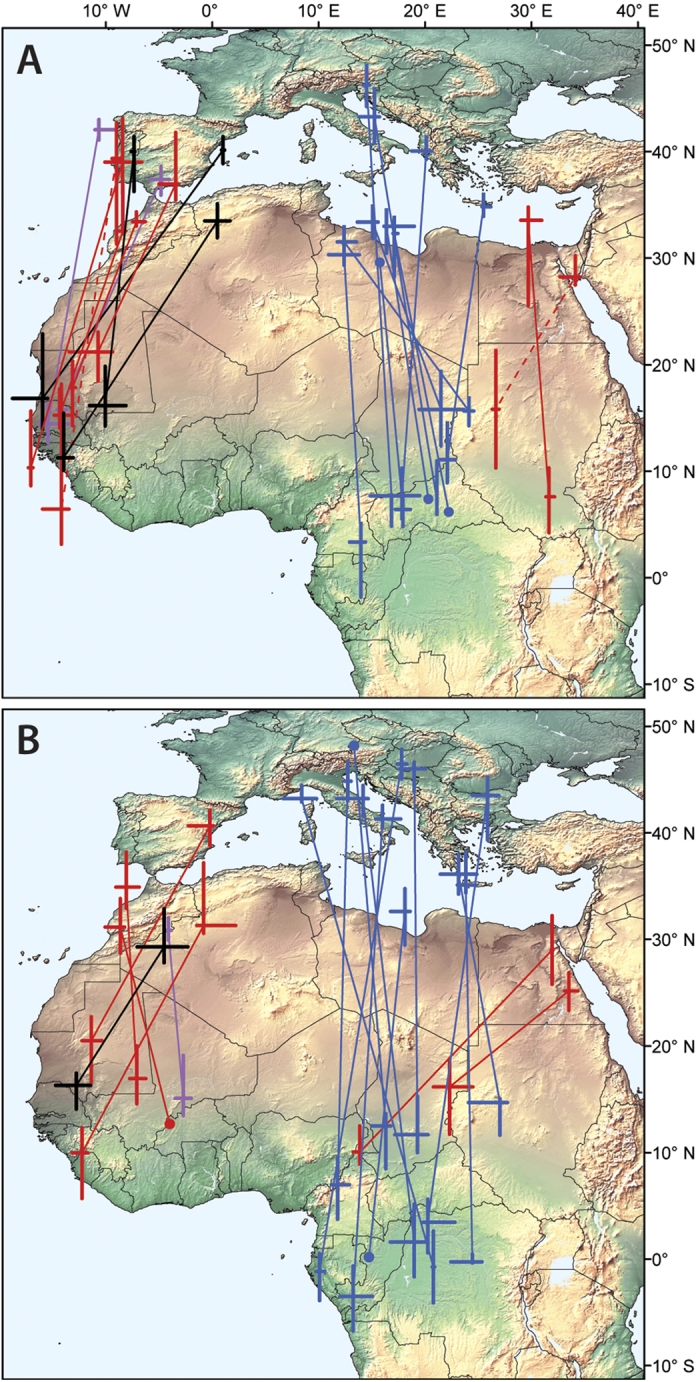
Autumn (**A**) and spring (**B**) stationary sites of birds (median ± 25^th^ and 75^th^ percentiles of location estimates) just prior and after the occurrence of the full light pattern (blue = collared flycatcher, black = pied flycatcher, red = Eurasian reed warbler, purple = aquatic warbler). The two dashed lines connect stationary sites for birds without FLP. The background map was made with Natural Earth public domain free vector and raster map data@naturalearthdata.com. The stationary sites were depicted in ArcGIS.

In all four species the occurrence of FLP coincides with the migration time between Europe and Africa (Supplementary Table S2). The majority of the stopover sites preceding and following FLPs are located North and South of the Sahara (Fig. 3), demonstrating that FLP occurred while the birds were crossing the Sahara and/or the Mediterranean Sea.

**Figure 4:**
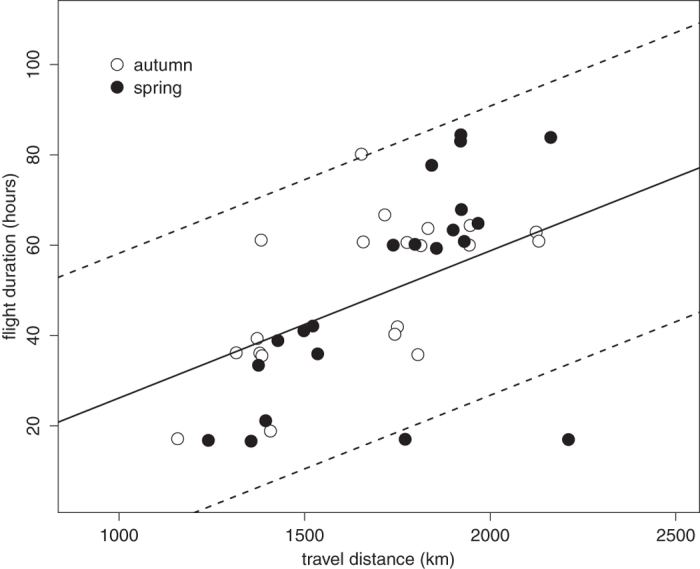
Relationship between travel distance (width of the Sahara desert on the individual crossing course) and the estimated duration of flight based on cumulative length of FLP (including night lengths). Fitted line ± 95% CI (dashed lines) is from a linear mixed-effect model. Note that there was no seasonal effect in the relationship (see Results). The point in the lower right corner was excluded as an outlier.

The estimated flight duration between stationary sites before and after the FLP was positively related to the estimated travel distance that the birds had to cross (b = 0.033 ± 0.009 SE, t = 3.9, P = 0.004; Fig. 4), while controlling for the non-significant effect of season (t = 1.5, P = 0.129; model means ± SE: autumn 52.1 ± 7.7 h, spring 47.7 ± 7.6 h; random effects variance: bird identity 49.3 (7.0 SD), species 170.4 (13.1 SD), residual variance 67.6 (8.2 SD)).

### Strategies of barrier crossing

In both seasons FLPs often ended abruptly during daytime on the last day of the presumed Sahara crossing (see middle plot of Fig. 1). We interpret this as prolonged flight into the day and the sudden change in light intensities as landing time (Fig. 2). In autumn FLP occurred in 73.5% (25 out of 34) of tracked birds and 47.1% (16) had an abrupt ending of FLP. Abrupt FLP endings mostly occurred on the last day and for the remaining birds various patterns were detected (Fig. 2). In spring all 23 tracked birds had FLP and abrupt ending occurred in 65.2% (15) of individuals, and as in autumn, the abrupt FLP ending occurred often on the last day (Fig. 2). We found no statistical difference in the number of FLPs occurring in autumn compared to spring (Chi-squared test with Yates’ correction χ^2^ = 0.34, df = 1, P = 0.561). Eight individuals prolonged their flights into the day during the two- or three-day period of barrier crossing but the durations of diurnal flights within individuals varied considerably (Fig. 2 and Supplementary Table S2). Four out of 22 birds tracked during both migratory periods showed the same pattern of barrier crossing (Supplementary Table S3). If we consider categories B and C (Fig. 2) as a variation on the same pattern, then 9 out of 22 birds showed the same pattern.

**Figure 5:**
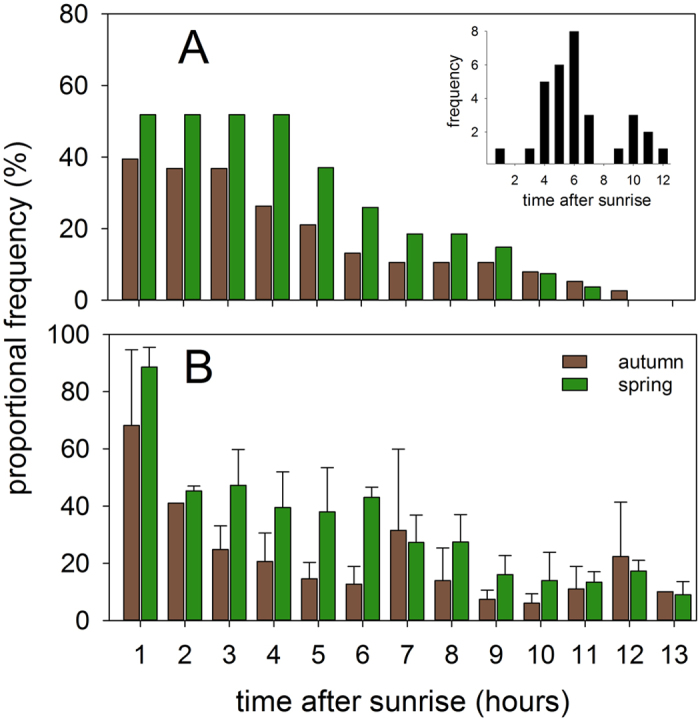
Estimates of proportions of birds with diurnal flights while crossing the Sahara desert and their timing of landing (inset in the upper right corner). The upper plot (**A**) shows frequencies of migratory flights into the day estimated from this study (excluding cases when T_max_ >92 min) and the lower panel (**B**) those (mean ± SD) recalculated from a radar field study in the Mauritanian Sahara^6^.

Consistent with radar measurements (Fig. 5b 6) we found FLP with T_max_ < 92 min (threshold time it took for each sunrise event to reach the maximum light intensity) and abrupt FLP endings to occur up to 12 hours after sunrise. The frequency of prolonged flights into the day (i.e. the number of FLPs with abrupt endings during daytime) was higher in spring (50% out of 28 cases) than in autumn (39.5% out of 38 cases; Fig. 5a) but this difference is not statistically significant (Kolmogorov-Smirnov test, D = 0.402, P = 0.31). The flights into the day ceased on average 5.8 ± 3.0 (SD) hours (n = 15 cases) after sunrise in autumn and 6.8 ± 2.1 hours (n = 14 cases) after sunrise in spring.

FLP without abrupt ending or any gaps by shading occurred in 26.5% (9) birds in autumn and 34.8% (8) birds in spring. Among them only one bird had perfect FLP and a low T_max_ on both FLP days in autumn (i.e. 2.9% of 34 checked individuals; pied flycatcher, T_max_ = 61 and 53 min) and another bird had perfect FLP for one day in spring (i.e. 4.3% of 23 individuals; aquatic warbler, T_max_ = 34 min). All remaining birds showed a combination of perfect FLP and gaps by shading.

Our simulation approach showed that, depending on the assumed flight speeds, between 20–80% of individuals fly into the day, to cover the distance for crossing the Sahara desert (Supplementary Fig. S3). The simulation shows that for spring migration a slightly higher percentage of daytime flights are necessary for crossing the desert (Supplementary Fig. S3).

## Discussion

High light intensities are typically recorded by geolocators in aerial foragers such as martins or swallows that stay airborne during large parts of the day (Fig. 1). In these “classic” diurnal migrants the light sensor is consistently exposed to the sun during the day. As a result, FLP is recorded regularly. We show here that four songbird species, which have been considered to be mainly nocturnal migrants, also show FLP during a limited migration period that coincides with their crossing of the Sahara desert. We suggest that migratory birds can flexibly switch from typical nocturnal migration to a prolonged flight into the day when facing the task of crossing a major ecological barrier. We found, however, that in three individuals (one aquatic warbler and two collared flycatchers) FLP was also detected outside the main migratory period (Supplementary Table S2). In two birds it occurred in November–December in sub-Saharan Africa (presumably effect of open habitat or perching behaviour) and in one bird at the breeding site in open habitat (marshes). Hence, at the moment we do not have any evidence for diurnal movements north of the Mediterranean.

In 9 individuals we did not find FLP while crossing the Sahara in autumn but for 7 of them we could still observe elevated light intensities that were above the average of typical light data recorded at times prior and after the desert crossing. We suggest that these birds behaved as typical nocturnal migrants that landed before dawn and rested during the day. In contrast, very few individuals (one in autumn and one in spring) showed perfect FLP either during the entire period of the Sahara crossing (or at least on some of the days needed for the crossing). Our very conservative suggestion is that these might be the non-stop flying individuals. An alternative view would be that the birds were resting on the ground without hiding in the shade. This seems unlikely, however, as the majority of observations of grounded birds show that they were actively hiding during daytime hours^17^. In addition, our simulation approach showed that the birds would have to fly at extreme speeds to cross the recorded distances by nocturnal flight only. Finally the diverse patterns of FLPs (Fig. 2) emphasise that birds are able to perform both diurnal and nocturnal migration even within one and the same journey.

The duration of FLP (i.e. the sum of nocturnal and diurnal periods) was positively related to the distance we calculated that the birds have to fly over the Sahara. For birds that crossed longer distances over the Sahara, multiple and/or longer durations of FLP were recorded. In contrast, birds that crossed the Sahara at its narrowest points had FLP usually only on one day. Interestingly, spring and autumn desert crossing times were similar. Hence it seems that desert crossing is optimized independently from seasonal time pressures; crossing the inhospitable region as fast as possible might be the main aim for every individual. And indeed, currently available geolocator studies showed fast crossing of the Sahara desert^7^,^18^,^19^.

Earlier field studies demonstrated that nocturnally migrating birds of unknown provenience prolong their flights into the day^3^,^11^,^20^,^21^. It was generally believed that these prolonged flights had just a very short duration and most birds tended to land shortly after dawn^6^,^11^,^22^ (but see^10^). Based on abrupt endings of the FLP during daytime, we found that the birds on average prolong the flights until noon during both autumn and spring migration. Our estimates of diurnal flights (as estimated from the durations of FLPs and T_max_ < 92 min) showed a gradual decline over the day. This pattern was remarkably similar to the one derived from the radar study in the western Sahara^6^ (recalculated in Fig. 5b). In spring, however, a higher proportion of tracked birds (i.e. with FLP and abrupt ending) prolonged their flights into the day than in autumn. Similarly, this seasonal pattern strongly resembled findings of the radar study by Schmaljohann *et al*.^6^. In addition, we found slightly higher occurrence of FLP in spring (25 out of 34 birds in autumn, while in all 23 individuals in spring). These two facts, however, do not match with estimates of desert crossing times which were similar for the two periods (see above). Faster total migration in spring is the general pattern across bird species^23^ but our data indicate that this might not be the case during desert crossing. That we found slightly more FLPs in spring might result from frequent tailwinds which prevail at higher flight altitudes at this time of the year^24^. Tailwinds are similar in autumn but at lower flight altitudes, in hotter and dry air^24^, which might explain the lower proportion of flights into the day. Accordingly, more birds prolonged their migration into the day under tailwind conditions as shown by radar^11^. Our data suggest that landing or searching for shade can occur nearly at any time of the day, most probably depending on when the bird reaches a suitable destination. This suggests an individually flexible prolongation of nocturnal migration into the day based on the bird’s needs and environmental conditions.

Another striking pattern we found was that the prolonged flights into day occurred most frequently on the last day of barrier crossing. Abrupt FLP endings were followed by much lower and variable light levels (i.e. a typical zigzag pattern) for the rest of the daytime than was typically observed on regular days without FLP. Field observations from the Sahara show that grounded fat birds (i.e. those that do not need to stop for refuelling) were often found resting in shade (e.g. single rocks, wadis, depressions or mountain ridges^4^,^17^) and our data seem to be in line with this. By simulation we estimated that depending on the flight speed between 20–80% of birds extended their nocturnal flights into the day in order to travel the distance they did.

To summarise, earlier studies detected diurnal flights of nocturnal migrants^3^,^9^,^10^ but were supposing that a small number of birds were doing so and that landing occurred shortly after dawn^6^. Here we emphasise that, at least in the four songbird species studied, prolonging flights into the day may be a common migratory pattern during barrier crossing. Such flights might be more common also in other barrier crossing systems, as has been recently shown for blackpoll warblers^13^. Based on the diverse patterns of FLP and its absence in some individuals, we emphasise the ability of birds to appropriately switch between diurnal and nocturnal migration when facing the challenge of crossing a large ecological barrier. There is accumulating evidence in larger-sized birds of considerable spatial but low temporal variability in migratory behaviour^25^,^26^,^27^. Often we found the same individuals to show different patterns of barrier crossing in autumn compared to spring journeys. This might be in line with the hypothesis of individually optimized migration schedules^28^. Such an assessment should be possible in the future by using larger data sets containing data from both sexes and repeated tracks of individuals across several migratory seasons.

## Methods

### Study species and the detection of light anomalies

From 2011 to 2012 individuals of four songbird species were equipped with geolocators (SOI-GDL 2.0, weight approx. 0.6 g, manufactured by the Swiss Ornithological Institute) at their European breeding grounds. After a year we retrieved 34 functional loggers: 13 from collared flycatchers, 4 from pied flycatchers, 12 from Eurasian reed warblers and 5 from aquatic warblers (Supplementary Table S2). The geolocators used an SMD photodiode EPD-470-1-0.9-1 (EPIGAP Optoelektronik, Germany) for light intensity measurements with a sensor wave length between 380 to 555 nm and a maximum range of about 3500 lux (corresponding to 63 arbitrary units). The SOI-GDL 2.0 geolocators recorded ambient light intensity in 5 min intervals.

The geolocators, conventionally used for positioning of migratory birds are also suitable for documenting changes in behaviour over the annual cycle^29^,^30^,^31^,^32^. When inspecting geolocator data in the four focal species we detected an obvious pattern of continuous full light intensity (hereafter full light pattern–FLP) with regular occurrence twice a year at times that coincide with the migratory period in many species (Fig. 1). We classified FLP as an uninterrupted period of >5 h (or >1 h on days with abrupt FLP ending, see below) during daytime where maximum light intensity (63 in arbitrary scale) was recorded.

An overview of individual FLP cases is given in Supplementary Table S2. The FLPs were classified into several categories in two steps based on a) the amount of shade of the daily light curves and b) time (T_max_) it took for each FLP sunrise event to reach the maximum light intensity (i.e. from 0/1 to 63 units). In the first step we fitted quadratic regressions to the sunrise data (delimited by the time of sunrise using the software Geolocator (SOI, Sempach) and the first consecutive data point which reached maximum light intensity) and sunset data (delimited by the last data point which reached maximum light intensity and sunset determined by the R-package GeoLight, version 1.03^33^) and summed up the absolute residuals. For the daytime period (delimited by the first and the last data point which reached maximum light intensity) we summed up all deviations from the maximal light intensity. These sums were used to assign every sunrise, day and sunset to the following categories: 1) perfect FLP, virtually no shading; 2) slight shading; 3) substantial shading. Additionally we assigned category 4 to FLPs with an abrupt start or end (Supplementary Fig. S1). In the second step, we calculated the T_max_ for each sunrise FLP event. We assumed that during the flight the bird was at an unknown height above ground. This implies no shading by vegetation or by folded wings occasionally covering the light sensor and thus a rapid increase in the recorded light intensity from 0 at twilight to maximum values. To extract sunrises for potential flights prolonged into the day from other sunrises, we compared the data to the sunrise pattern recorded by a typical diurnal migrant and aerial forager. We used light-level logger data of barn swallows *Hirundo rustica* breeding in southern Switzerland and migrating along the central European-African flyway^34^. We selected 6 days during autumn (n = 10 birds) and spring (n = 7 birds) migration, when the birds moved between 16° and 35°N (southern borders of the Sahel and the Mediterranean Sea) and vice versa. This was at periods between 12–30 Sept and 10 March–17 April. The maximum T_max_ value and its 95^th^ percentile in barn swallows were 127 min and 91 min, respectively (Supplementary Fig. S2). The latter was used as a threshold for our conservative estimates of prolonged flights into the day. Hence, unless otherwise stated, for further analyses of flight into the day we considered only those FLP cases when T_max_ < 92 min (68 FLP events, 27 excluded) and the FLP was classified as 1, 2 or 4.

### Determining stationary periods

Data from autumn and spring were analysed independently using January 1 as a separator. We calculated stationary periods prior and after the occurrence of FLP using the changeLight function of the R-package GeoLight with minimum staging period set at 3 days. We filtered outlying positions that were >800 km from the median latitude of a given stationary site. We defined a stationary site to be the median of the geographic coordinates ± their 25^th^/75^th^ percentiles within the particular stationary period. The same number of interquartile ranges (k = 2) of the loessFilter function was used for all individuals of the same species except for one bird (7OY, k = 1.1). To determine the first stationary period before and after the FLP the probability threshold of the changeLight function was adjusted for each bird individually. For autumn, geographic positions of the stationary periods before and after the FLP were calculated using sun-elevation angles derived from the in-habitat calibration in the breeding areas or Hill-Ekstrom calibration from data of the respective stationary period^35^. When one of the calibration techniques was not applicable or failed, the other was used instead. Please note that we were not able to determine stationary sites for all birds (available estimates are for 22 birds in autumn and 19 in spring). An example of light data profile used to determine the stationary periods before and after crossing the barrier is provided in Supplementary Fig. S4.

### Duration of light anomalies

We considered two scenarios for estimating the duration of potential flight over the Sahara Desert at times when FLP occurred: nocturnal flight only or including prolonged flight into the daytime. When FLP ended abruptly during daytime, we took that abrupt change (accuracy to 5 min) as a termination of FLP and the assumed prolonged flight. We estimated the theoretical duration of the prolonged flight as nocturnal flight plus FLP. We assumed that the bird took off for the flight within an hour after sunset the day preceding the occurrence of FLP^35^,^36^,^37^,^38^. For cases when there were two or more periods of FLP separated by days without FLP, we excluded the daytime non-FLP period from the estimates of flight times. In those cases when an abrupt end of FLP occurred during the day, we added the time period between sunrise and the moment of abrupt decline of light data to the nocturnal flight duration. For cases without an abrupt end of FLP, landing time was estimated to be within an hour before the sunrise on the day that followed the FLP day^11^. Duration of nocturnal flights only was estimated as a sum of night lengths before, during and after the FLP.

We compared the frequency of potential flights into day based on abrupt FLP endings with those found in an empirical study provided by^6^. We recalculated the migration traffic rates from their original dataset by setting nocturnal migration traffic rates to 100 and calculated the declining proportion of traffic rates binned to hours after sunrise in autumn and spring.

### Flight range estimates during FLP times

Distance between stationary sites was measured as the loxodromic distance between median positions of the last stationary site before the FLP and the first thereafter. For an approximation of barrier crossing distances, we estimated the width of the Sahara desert (minimum travel distance) at points where the bird presumably entered and exited the desert on the loxodromic line that connects the stationary sites just before and after the FLP. Northern and southern desert borders were derived from the land cover map from the GLC2000 database, European Commission Joint Research Centre, http://bioval. jrc.ec.europa.eu/products/glc2000/glc2000.php. We hypothesized that the duration of FLP was driven by the width of the desert and the barrier-crossing strategy of an individual. The relationship between travel distance and the estimated duration of flight during FLP was assessed by a linear mixed-effect model in the R-package lme4. We ran a model with flight duration as response variable that included our estimates of summed time for both nocturnal and diurnal migration (n = 40 cases after excluding one case, a reed warbler where a distance of 2211 km in 17 h was considered as an outlier, see Fig. 4. This individual would have to fly at speed of ca 130 km h^−1^ which is very unlikely). The fixed effect was travel distance, while season (autumn, spring) was taken as a covariate. Individual identity nested within species was entered as a random effect. We obtained similar results (not shown) when we ran the same analysis with travel distances between the stationary sites. All data analyses were conducted in R version 3.0.1^39^.

### Ethical note

The field work was carried out in accordance with the current laws of Belarus, Czech Republic, Finland, Germany, Sweden and Ukraine. The procedures used to handle and fit the birds with geolocators were approved by Academy of Sciences of the Czech Republic (#38/2011), Varsinais-Suomi Centre for Economic Development, Transport and the Environment (#LOS-2009-L-308-259), Landratsamt Saale-Orla-Kreis (#16.075.364.622.0 SC/12), Landkreis Leipzig (364.620/15/7/4), Stockholms södra djurförsöksetiska nand (#S55-11), Ukrainian Ministry of Ecology and Natural Resources (1/2011) and by ethical committees of Palacký University and Czech Ministry of Education (#1/2011, licence #CZ00231).

## Additional Information

**How to cite this article**: Adamík, P. *et al*. Barrier crossing in small avian migrants: individual tracking reveals prolonged nocturnal flights into the day as a common migratory strategy. *Sci. Rep.* **6**, 21560; doi: 10.1038/srep21560 (2016).

## Supplementary Material

Supplementary Information
