# Supplementary material for: Barrier crossing in small avian migrants: individual tracking reveals prolonged nocturnal flights into the day as a common migratory strategy
Source: Sci Rep. 2016 Feb 15;6:21560. doi: 10.1038/srep21560 (PMC4753512; doi:10.1038/srep21560)
Supplement: Supplementary Information [file srep21560-s1.pdf]

Supplementary Online Materials

Barrier crossing in small avian migrants: individual tracking reveals prolonged nocturnal flights into the day as a common migratory strategy

Peter Adamík[1, 2]*†, Tamara Emmenegger[3]†, Martins Briedis[1]†, Lars Gustafsson[4], Ian Henshaw[4], Miloš Krist[1, 2], Toni Laaksonen[5], Felix Liechti[3], Petr Procházka[6], Volker Salewski[7], Steffen Hahn[3]†

[1]Department of Zoology, Palacký University, tř. 17. listopadu 50, CZ-771 46 Olomouc, Czech Republic

[2]Museum of Natural History, nám. Republiky 5, CZ-771 73 Olomouc, Czech Republic

[3]Department of Bird Migration, Swiss Ornithological Institute, Seerose 1, CH-6204 Sempach, Switzerland

[4]Department of Animal Ecology/Ecology and Genetics, Uppsala University, Norbyvägen 18D, SE-75236, Uppsala, Sweden

[5]Section of Ecology, Department of Biology, University of Turku, FI-20014, Turku, Finland

[6]Institute of Vertebrate Biology, Academy of Sciences of the Czech Republic, Květná 8 ,CZ-603 65 Brno, Czech Republic

[7]Michael-Otto-Institut im NABU, Goosstroot 1, D-24861 Bergenhusen, Germany

* To whom correspondence should be addressed. E-mail: peter.adamik@upol.cz

Table S1. Classification of long-distance migratory birds according to their migratory strategy: either nocturnal (N; birds rest during the day and fly at night) or diurnal (D; resting during the night and flying at daytime). When mixed strategies exist the less prevalent strategy is given in parentheses. Listed are species for which at least a part of European breeding population winters S of the Sahara Desert. This non-exhaustive compilation includes only species with reliable information and it relies mainly on expert knowledge, basic handbook sources and information from a long-term ringing monitoring in the Alps (Col de Bretolet) run by the Swiss Ornithological Institute.

Common name	Scientific name	Migratory strategy
Great Reed Warbler	*Acrocephalus arundinaceus*	N
Aquatic Warbler	*Acrocephalus paludicola*	N
Marsh Warbler	*Acrocephalus palustris*	N
Sedge Warbler	*Acrocephalus schoenobaenus*	N
Eurasian Reed Warbler	*Acrocephalus scirpaceus*	N
Tawny Pipit	*Anthus campestris*	D & N
Red-throated Pipit	*Anthus cervinus*	D
Tree Pipit	*Anthus trivialis*	D & N
Common Swift	*Apus apus*	D & N
Alpine Swift	*Apus melba*	D & N
Pallid Swift	*Apus pallidus*	D & N
Greater Short-toed Lark	*Calandrella brachydactyla*	D & N
European Nightjar	*Caprimulgus europaeus*	N
Red-rumped Swallow	*Cecropis daurica*	D
Rufous-tailed Scrub Robin	*Cercotrichas galactotes*	N
Great Spotted Cuckoo	*Clamator glandarius*	D & N
European Roller	*Coracias garrulus*	D
Common Quail	*Coturnix coturnix*	N
Common Cuckoo	*Cuculus canorus*	(D) & N
Cream-colored Courser	*Cursorius cursor*	D
Common House Martin	*Delichon urbicum*	D
Ortolan Bunting	*Emberiza hortulana*	D & N
Collared Flycatcher	*Ficedula albicollis*	N
European Pied Flycatcher	*Ficedula hypoleuca*	N
Semi-collared Flycatcher	*Ficedula semitorquata*	N
Black-winged Pratincole	*Glareola nordmanni*	D
Collared Pratincole	*Glareola pratincola*	D
Icterine Warbler	*Hippolais icterina*	N
Melodious Warbler	*Hippolais polyglotta*	N
Barn Swallow	*Hirundo rustica*	D
White-throated Robin	*Irania gutturalis*	N
Red-backed Shrike	*Lanius collurio*	N
Isabelline Shrike	*Lanius isabellinus*	N
Lesser Grey Shrike	*Lanius minor*	N
Woodchat Shrike	*Lanius senator*	N
River Warbler	*Locustella fluviatilis*	N
Savi's Warbler	*Locustella luscinioides*	N
Common Grasshopper Warbler	*Locustella naevia*	N

Thrush Nightingale	*Luscinia luscinia*	N
Common Nightingale	*Luscinia megarhynchos*	N
Bluethroat	*Luscinia svecica*	N
European Bee-eater	*Merops apiaster*	D & N
Rufous-tailed Rock Thrush	*Monticola saxatilis*	N
Blue Rock Thrush	*Monticola solitarius*	N
White Wagtail	*Motacilla alba*	D & N
Grey Wagtail	*Motacilla cinerea*	D
Western Yellow Wagtail	*Motacilla flava*	D & N
Spotted Flycatcher	*Muscicapa striata*	N
Desert Wheatear	*Oenanthe deserti*	N
Black-eared Wheatear	*Oenanthe hispanica*	N
Northern Wheatear	*Oenanthe oenanthe*	N
Eurasian Golden Oriole	*Oriolus oriolus*	(D) & N
Common Redstart	*Phoenicurus phoenicurus*	N
Western Bonelli's Warbler	*Phylloscopus bonelli*	N
Common Chiffchaff	*Phylloscopus collybita*	D & N
Iberian Chiffchaff	*Phylloscopus ibericus*	N
Wood Warbler	*Phylloscopus sibilatrix*	N
Willow Warbler	*Phylloscopus trochilus*	N
Eurasian Crag Martin	*Ptyonoprogne rupestris*	D
Sand Martin	*Riparia riparia*	D
Whinchat	*Saxicola rubetra*	N
Eurasian Stonechat	*Saxicola torquatus*	N
Eurasian Blackcap	*Sylvia atricapilla*	N
Garden Warbler	*Sylvia borin*	N
Subalpine Warbler	*Sylvia cantillans*	N
Common Whitethroat	*Sylvia communis*	N
Lesser Whitethroat	*Sylvia curruca*	N
Orphean Warbler	*Sylvia hortensis*	N
Barred Warbler	*Sylvia nisoria*	N
Eurasian Hoopoe	*Upupa epops*	D & (N)

Table S2. Patterns of occurrence of FLP during autumn and spring migration. **Breeding population** gives the European breeding site locations, **Migratory period** refers whether FLP occurred in spring or autumn, **Dates of light anomaly (FLP)** listed are days when a distinct light pattern occurred that could be interpreted as FLP or as a strong zigzagging pattern potentially indicating that the bird was in the desert but resting in shelter during the daytime**, # days with FLP** gives the number of days with FLP, **Abrupt FLP ending** (yes or no) reports for each case whether FLP ended abruptly during the daytime**, Elapsed time** gives the time in hours between sunrise and the abrupt end of FLP during the daytime, i.e. the duration of prolonged flight into the day. **T_{max}** gives the time (in minutes) it took to reach the maximum light intensities. Values that were over the threshold of 91 min are in bold. Note that spring and autumn tracks are not available for all individuals as in several cases the geolocator battery life span was less than a year. In three individuals (7EN, 7HO and 7JA) light anomalies (> 5h of max light intensities) were also recorded on several occasions outside of the main migration period, see remarks at the bottom of the table.

Species	Logger ID	Breeding population	Migratory period	Dates of FLP	# days with FLP	Abrupt FLP ending	Elapsed time	T_{max}
Reed Warbler	7QN	Germany	autumn	7-11 October 2012***	0			
Reed Warbler	7RY	Germany	autumn	8 October 2012	1	yes	6.4	42
Reed Warbler	7RJ	Germany	autumn	26-28 September 2012***	0			
Reed Warbler	7QK	Germany	autumn	-	0			
Reed Warbler	9CI	Germany	autumn	13 September 2012	1	yes	5.5	62
Reed Warbler	7QG	Germany	autumn	-	0			
Reed Warbler	9BI	Czech Republic	autumn	22-24 September 2012***	0			
Reed Warbler	7SF	Czech Republic	autumn	31 Aug, 18-19 Sept 2012***	0			
Reed Warbler	7SC	Czech Republic	autumn	14-15 September 2012***	0			
Reed Warbler	9BL	Czech Republic	autumn	10 September 2012	1			73
Reed Warbler	9AZ	Czech Republic	autumn	1 September 2012	1	yes**		**347**
Reed Warbler	9BV	Czech Republic	autumn	12 September 2012***	0			
Reed Warbler	7QN	Germany	spring	13-14 April 2013	2	yes, yes	9.8, 6.6	43, 47
Reed Warbler	7QK	Germany	spring	18 May 2013	1	yes	6.1	91
Reed Warbler	9CI	Germany	spring	11-12 April 2013	2	yes, yes	10.2, 5.2	42, 52
Reed Warbler	7QG	Germany	spring	3 May, 5 May 2013	1+0+1	yes, yes	6.8, 3.6	62, 82
Reed Warbler	9BI	Czech Republic	spring	15 May 2013	1	yes	6.3	85

Reed Warbler	7SC	Czech Republic	spring	13 May 2013	1	yes	10.5	47
Reed Warbler	9AZ	Czech Republic	spring	14 March 2013	1	yes	4.8	58
Reed Warbler	9BV	Czech Republic	spring	25 March 2013	1	yes	5.5	**137**
Pied Flycatcher	3GS	Finland	autumn	16-17 September 2011	2	yes	5.5	**167, 202**
Pied Flycatcher	3JE	Finland	autumn	30 Sept-1 October 2011	2			61, 53
Pied Flycatcher	3OX	Finland	autumn	5-6 October 2011	2			**127, 98**
Pied Flycatcher	3PG	Finland	autumn	5-6, 8 October 2011	2+0+1	no, yes	7.3	74, 73, **92**
Pied Flycatcher	3GS	Finland	spring	11 April 2012	1	yes	7.9	**212**
Pied Flycatcher	3JE	Finland	spring	17-18 April 2012	2	yes	5.1	64, 78
Collared Flycatcher	3AE	Czech Republic	autumn	29-30 August 2011	2			38, 63
Collared Flycatcher	3EU	Czech Republic	autumn	7-9 September 2011	3	yes	3.6	53, 37, 42
Collared Flycatcher	7HA	Sweden	autumn	9-10 September 2012	2	yes	5.4	**203, 132**
Collared Flycatcher	7HN	Sweden	autumn	8-9 September 2012	2			**123**, 81
Collared Flycatcher	7HO	Sweden	autumn	18-20 September 2012	3	yes	4.1	52, 32, 37
Collared Flycatcher	7IB	Sweden	autumn	16-17 September 2012	2			57, 54
Collared Flycatcher	7IG	Sweden	autumn	22-23 September 2012***	0			
Collared Flycatcher	7JA	Sweden	autumn	7-8 September 2012	2			62, 42
Collared Flycatcher	7JB	Sweden	autumn	22-23 September 2012	2	yes	8.3	**127, 127**
Collared Flycatcher	7JI	Sweden	autumn	21-23 September 2012	3	yes, yes	6.9, 6.0	40, 39, 37
Collared Flycatcher	7JJ	Sweden	autumn	21-23 September 2012	3	yes, yes	5.5, 3.8	**112**, 55, 62
Collared Flycatcher	7LA	Sweden	autumn	19-20 September 2012	2	yes	4.8	57, 62
Collared Flycatcher	7LH	Sweden	autumn	18-19 September 2012	2			**147**, 69
Collared Flycatcher	3EU	Czech Republic	spring	25-27 April 2012	3			58, 85, **93**
Collared Flycatcher	7HN	Sweden	spring	6-8 April 2013	3	yes	4.5	74, 73, **93**
Collared Flycatcher	7HO	Sweden	spring	7-9 April 2013	3			77, 74, 57
Collared Flycatcher	7IB	Sweden	spring	13-14, 16 April 2013	2+0+1	no, yes	5.2	57, 62, 52
Collared Flycatcher	7IG	Sweden	spring	11-13 April 2013	3	yes, yes	7.9, 9.2	82, 72, **157**
Collared Flycatcher	7JA	Sweden	spring	25-27 April 2013	3			43, **93**, 69
Collared Flycatcher	7JB	Sweden	spring	22-23 April 2013	2			82, **142**
Collared Flycatcher	7JI	Sweden	spring	23-24 April 2013	2			**147, 132**
Collared Flycatcher	7JJ	Sweden	spring	22-23 April 2013	2			72, 88

Collared Flycatcher	7LA	Sweden	spring	1-3 May 2013	3	yes	8.2	**119**, 78, **102**
Collared Flycatcher	7LH	Sweden	spring	22-23 April 2013	2			**172, 143**
Aquatic Warbler	7EN	Belarus	autumn	multiple periods over the annual cycle	*			
Aquatic Warbler	7OY	Ukraine	autumn	26-27 August 2012	1+1	yes, yes	11.6, 2.5	38, 47
Aquatic Warbler	7PE	Belarus	autumn	21 Aug, 29-30 Aug 2012	1+0+2	no, yes	10.4	**107**, 32, 39
Aquatic Warbler	7PO	Ukraine	autumn	23-24 August 2012	2	yes	1.0	38, 44
Aquatic Warbler	7HK	Belarus	autumn	18-19 August 2012	1+1	yes, yes	11.1, 4.4	47, 43
Aquatic Warbler	7EN	Belarus	spring	9 March 2013	1			34
Aquatic Warbler	7OY	Ukraine	spring	26 March 2013	1	yes	5.7	62

* a case that cannot be clearly interpreted as FLP occurred multiple times but we could not estimate the stationary sites around those FLP days; ** unusual FLP pattern when low light intensities occurred in the morning followed by FLP for the rest of the day; *** in these cases FLP was absent but on those days we could still observe elevated but zigzagged light recordings during daytime.

Light anomalies were detected in three individuals also during other times when they were presumably not crossing any barriers: 7HO – on 16 Nov 2012 and 22 Nov 2012, both cases with FLP only for several hours during the middle of the day and with considerable shading effect, 7JA – 6 cases of FLP between 15 Nov and 4 Dec 2012, 7EN – a series of 11 days with FLP of various quality between 15 August 2012 and 23 Sept 2012 (we assume that some of them were during barrier crossing but we fail to estimate stationary sites around the FLP days) and another series with 8 FLPs days of various quality between 11 May 2013 and 19 May 2013.

Table S3. A comparison of autumn and spring migratory strategies used by individual birds that were tracked during the entire annual cycle. Strategies, listed by capital letters, are from Fig. 2. Note that categories C and B are from the perspective of barrier crossing likely identical.

**Species**	**Logger ID**	**Category of light pattern** Autumn	Spring
Pied Flycatcher	3GS	C*	C
Pied Flycatcher	3JE	B	C
Collared Flycatcher	3EU	C	B
Collared Flycatcher	7HN	B	C
Collared Flycatcher	7HO	C	B
Collared Flycatcher	7IB	B	G
Collared Flycatcher	7IG	A	D
Collared Flycatcher	7JA	B	B
Collared Flycatcher	7JB	C	B
Collared Flycatcher	7JI	D	B
Collared Flycatcher	7JJ	F	B
Collared Flycatcher	7LA	C	C
Collared Flycatcher	7LH	B	B
Aquatic Warbler	7OY	E	C
Aquatic Warbler	7EN	not interpretable	B
Reed Warbler	7QG	A	G
Reed Warbler	7QK	A	C*
Reed Warbler	7QN	A	E
Reed Warbler	7SC	A	C*
Reed Warbler	9AZ	H	C*
Reed Warbler	9BI	A	C*
Reed Warbler	9BV	A	C*
Reed Warbler	9CI	C*	E

* cases when FLP occurred during one day only and the bird landed during the daytime

Fig. S1. Illustrative examples of FLP classification based on shadiness. From the whole FLP (first left column) the sunrise (second column) and sunset (fourth column) have been classified using the sums of the absolute residuals from a fitted quadratic regression when light (y-axis) is plotted against time (x-axis, red line). The daytime period (third column) was classified using the sum of all deviations from the maximum light intensity (red line = 63 units). The bottom row shows data with considerable shading early in the morning. Such FLP cases were excluded from our interpretation as flights into the day.

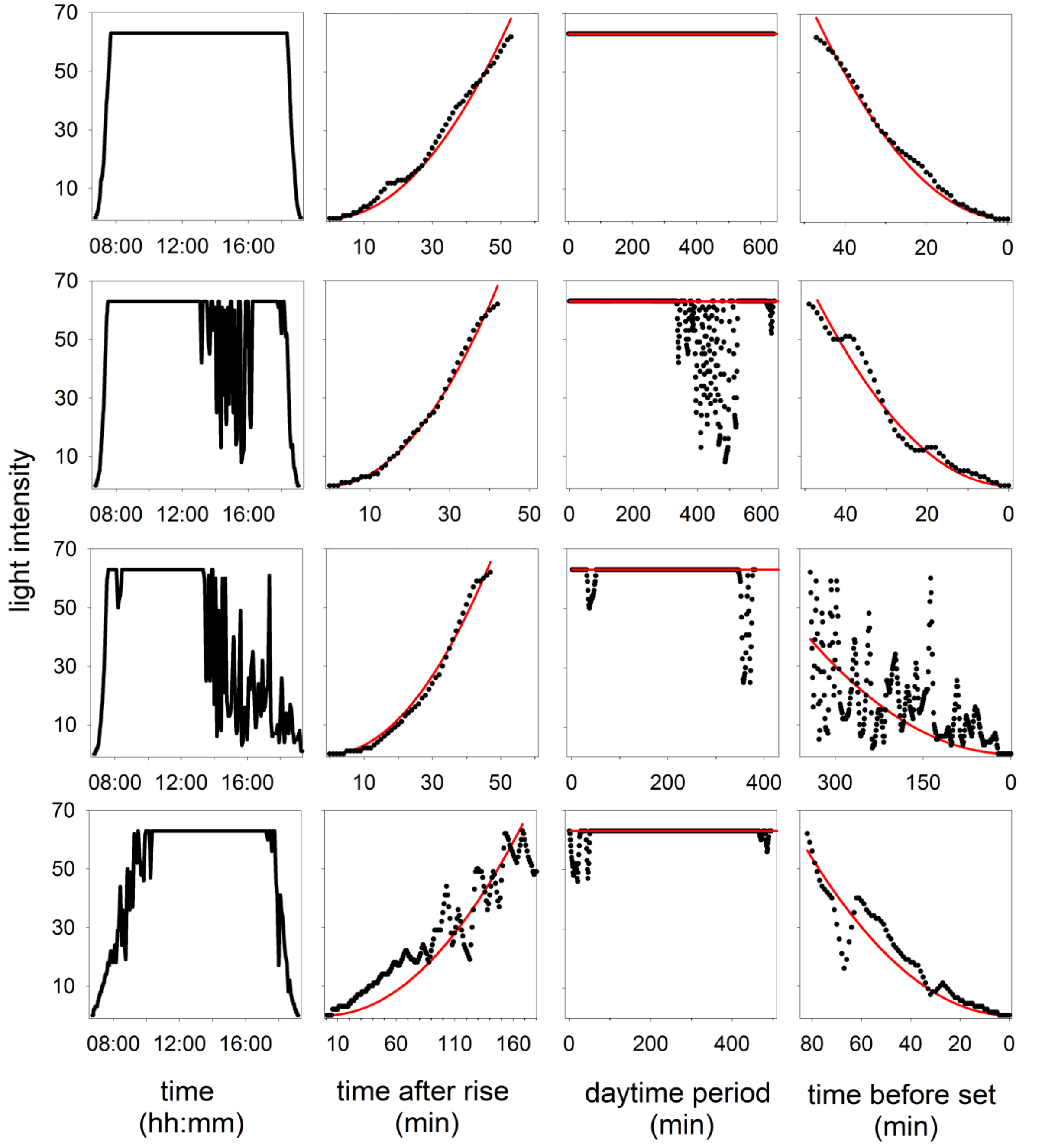

Fig. S2. Distribution of T_{max} values for barn swallows (black bars – test data) and our four focal species (grey bars – FLP data).

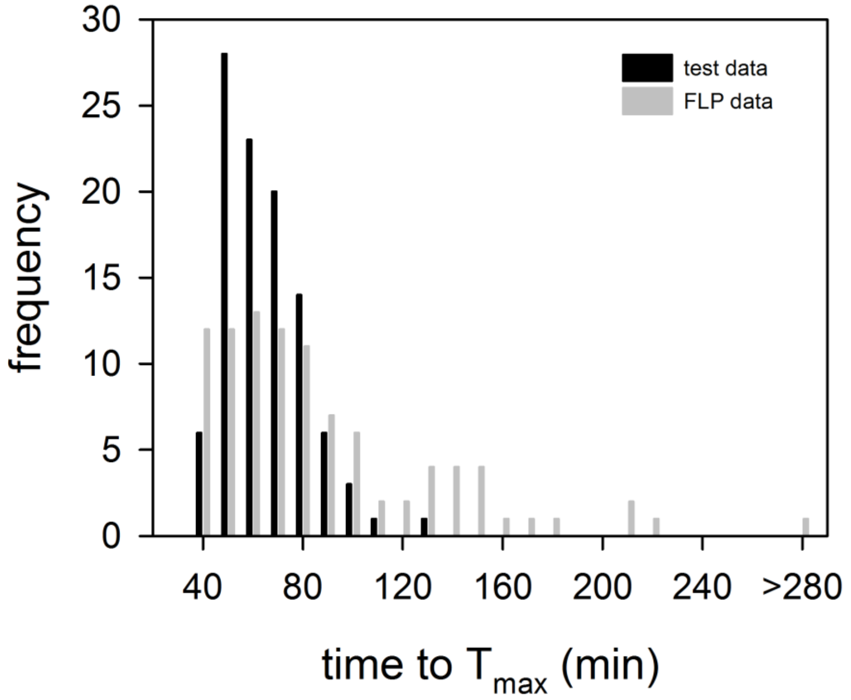

Fig. S3. Simulation (separately for autumn and spring data) of how many individuals (bars in %) in our study had to fly at least partly into the day in order to be able to cross the desert under three empirically measured flight speeds. The average ground speeds were taken as 50 km h^{-1} (Schmaljohann, Liechti, and Bruderer 2007) , 59 km h^{-1} (Salewski, Schmaljohann, and Liechti 2010) and 76 km h^{-1} (Biebach et al. 2000). We used these speeds and the travel distance (the width of the Sahara each individual had to cross – see Methods) to calculate the time the bird must be aloft. Then we compared this time estimate with the summed nocturnal flight times from the night preceding the FLP, all nights between FLP days and the night after the last FLP day if we did not find an abrupt ending. Total time (summed during FLP; circles in hours on right vertical axis) of expected flight into the day ranged between 6h at 50 km h^{-1} to max 16h for the spring model at 59 km h^{-1} speed (median ± 25/75 percentiles). The lower panel gives the frequencies of total flight times into the day (binned to 2 hours), which decreased with higher ground speeds.

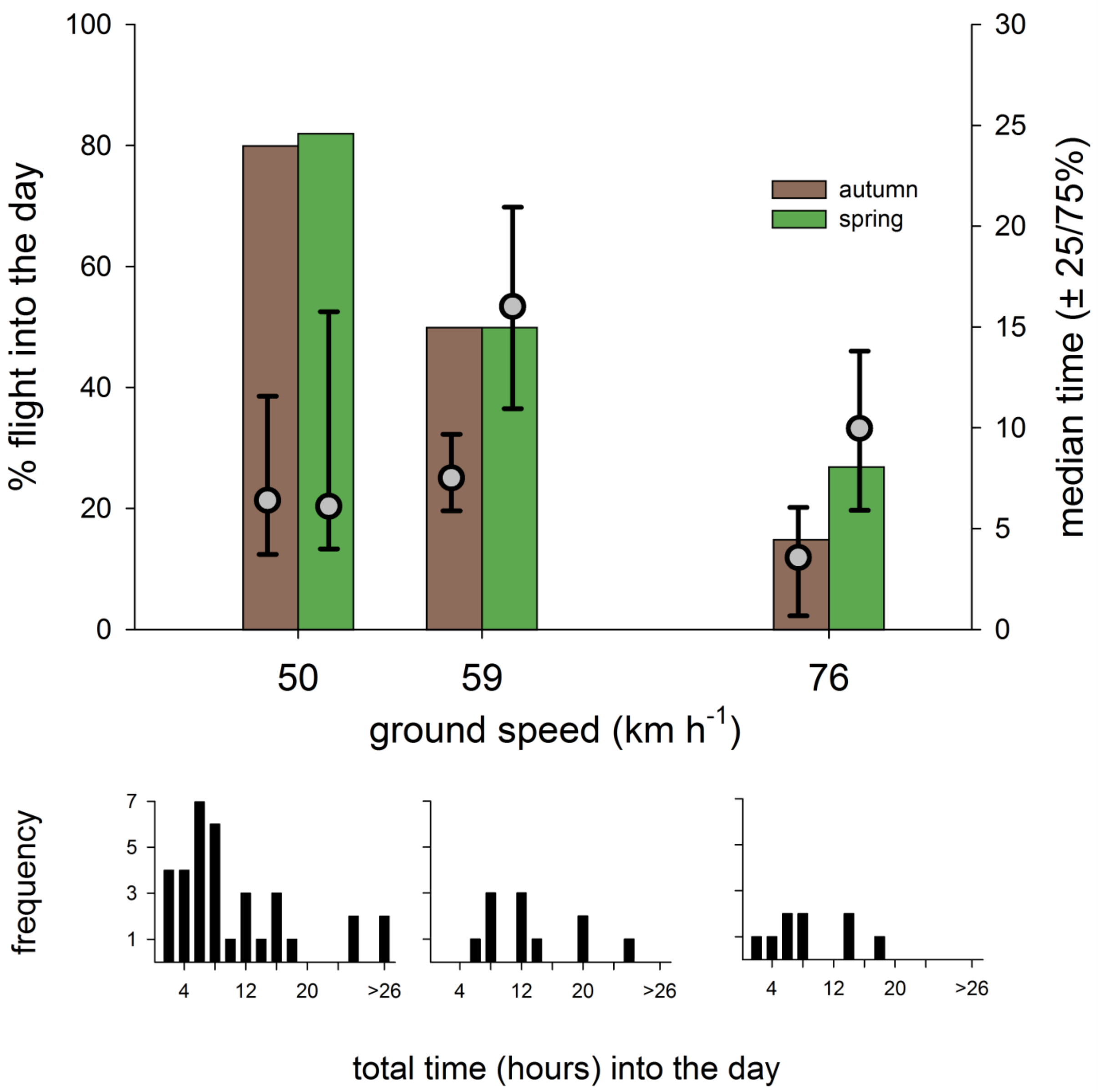

Fig. S4. A representative example of light data profile (collared flycatcher #7HO during autumn migration) used to determine the stationary periods before and after crossing the barrier. The FLP anomaly is in grey area.

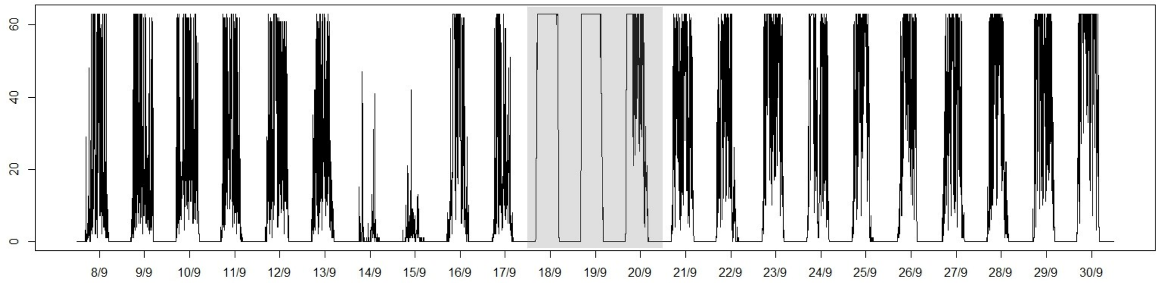

References

Biebach H, Biebach I, Friedrich W, Heine G, Partecke J, Schmidl D. 2000. Strategies of passerine migration across the Mediterranean Sea and the Sahara Desert: a radar study. Ibis 142:623–634.

Salewski V, Schmaljohann H, Liechti F. 2010. Spring passerine migrants stopping over in the Sahara are not fall-outs. J. Ornithol. 151:371–378.

Schmaljohann H, Liechti F, Bruderer B. 2007. Songbird migration across the Sahara: the non-stop hypothesis rejected! Proc. R. Soc. B-Biological Sci. 274:735–739.
